# Ageing with a silver‐spoon: A meta‐analysis of the effect of developmental environment on senescence

**DOI:** 10.1002/evl3.79

**Published:** 2018-08-16

**Authors:** Eve B. Cooper, Loeske E. B. Kruuk

**Affiliations:** ^1^ Division of Ecology and Evolution, Research School of Biology The Australian National University Acton Canberra ACT 2601 Australia

**Keywords:** Ageing, actuarial senescence, bird, developmental programming, environmental conditions, mammal, meta‐analysis, phenotypic plasticity, reproductive senescence, silver‐spoon

## Abstract

What determines variation between individuals in how they senesce, and are environmental conditions experienced during development relevant to late‐life performance? We report a meta‐analysis of studies of wild populations to determine how the quality of the environment experienced during development affects rates of survival and reproductive senescence. From studies of 14 bird or mammal species, we calculated effect sizes for the interaction between the effects of environmental quality during development and age in predicting survival (*N* = 18) or reproduction (*N* = 30) over time in late life. We found no evidence that developmental environment affected rates of survival senescence (β_mean_ = –1.2 × 10^−4^ ± 0.022SE). However, a better developmental environment was associated with slower rates of reproductive senescence in late life (β_mean_ = 0.062 ± 0.023SE), indicating a small, but significant, “silver‐spoon” effect of early‐life conditions that persisted through to late life. Our results illustrate how the effects of environmental conditions during development can persist throughout life, and indicate one possible cause of phenotypic plasticity in senescence.

**Impact summary**

Can the quality of the environment experienced during the first stage of life, the developmental period, have persistent effects that last into the final stage of life, affecting rates of senescence? Senescence, or the decline in reproduction or survival in old age, is common in both wild and domestic populations, but may also vary substantially between individuals in a population for different environmental and genetic reasons. Several recent analyses of data from detailed long‐term studies of wild animal populations have now tested the effect of developmental environment on senescence, across a range of species. Here, we used a meta‐analysis of these studies to summarize the effect of developmental environmental quality on senescence rates in the wild. Across 14 species of birds and mammals, there was no evidence that developmental environment influenced survival senescence; however, there was a significant influence on reproductive senescence. Being exposed to a higher quality environment during the developmental period provided a “silver‐spoon,” whereby individuals experienced slower declines in reproductive ability at the very last stage of their lives. Our results illustrate how the very first phase of life can shape the very last phase of life.

Long‐standing evolutionary questions surround the ultimate causes and consequences of senescence, defined as the decline in fitness‐related traits with age (Medawar 1952; Williams 1957; Kirkwood and Austad 2000). One aspect of an individual's senescence pattern, their rate of senescence, is measurable as the rate of age‐related decline between the onset of senescence and death. Declines in survival probability and reproductive output (coined “survival” or actuarial, senescence, and “reproductive” senescence, respectively) are the most commonly used metrics of senescence rates (Lemaître and Gaillard 2017), likely because they correspond to direct components of individual fitness (Bouwhuis et al. 2012; Kowald and Kirkwood 2015). Increasing numbers of longitudinal wild animal studies have now demonstrated that variation in individual senescence rates even within populations is common in natural environments (Nussey et al. 2013). In determining the evolutionary causes and consequences of senescence, it is therefore important to understand what causes these interindividual differences. In essence, what determines how fast, or how slow, an individual will senesce?

The extrinsic environment may play a key role in shaping rates of senescence in both reproduction and survival (Kawasaki et al. 2008; Nussey et al. 2013; Lemaître and Gaillard 2017). Populations of the same species exposed to different environments often experience widely different patterns of senescence (Austad 1993; Lemaitre et al. 2013; Douhard et al. 2016; Holand et al. 2016). On an evolutionary scale, differences in senescence patterns observed between populations could be caused by natural selection, whereby differences in extrinsic mortality risk cause differential selection pressures on senescence rates (Medawar 1952; Williams 1957). Conversely, the quality of the environment experienced in late‐life has a direct effect on senescence, with better environmental conditions typically being associated with slower senescence (Reichert et al. 2010; Pardo et al. 2013; Oro et al. 2014; Arlet et al. 2015; Bleu et al. 2015; Chantepie et al. 2016). Additionally, senescence rates can theoretically be influenced by the extrinsic environment not only at the time when senescence occurs, but at any point in an individual's life‐history. It is therefore conceivable that the earliest stage of life, the developmental period, could influence an individual's senescence rates.

The theory of developmental programming suggests that the period of development, which includes both gestation and juvenility up until the point of sexual maturity, is distinct in its role of modulating the adult phenotype (Lindström 1999; Hales and Barker 2001; Monaghan 2008). Developmental programming first gained prominence in a series of seminal studies on human health where it was found that an apparent poor developmental environment is strongly associated with increased risk of impaired glucose tolerance (Hales et al. 1991), type two diabetes (Hales and Barker 1992), and cardiovascular disease (Barker et al. 1993) later in life. Since then, it is been demonstrated that poor developmental conditions in humans is also associated with earlier menopause (Elias et al. 2003; Tom et al. 2010), reduced longevity (Modin et al. 2009; Todd et al. 2017), and even behavioral shifts reflecting a faster pace‐of‐life (Mell et al. 2018).

Among nonhuman animals, exposure to good environmental conditions during development, such as high nutritional availability, can have generally positive impacts on a multitude of aspects of individual fitness beyond the developmental period itself (see Lindström 1999 for a review). Grafen (1988) referred to this phenomenon as the “silver‐spoon” effect. Although there is now abundant evidence for the silver‐spoon effect on performance in early and mid‐life in wild animals, what is not yet established is what influence the environment experienced during the developmental period has on late life, and specifically on senescence (Nussey et al. 2013; Lemaître et al. 2015; Lemaître and Gaillard 2017). If silver‐spoon effects do extend to the last stage of life, a better quality developmental environment is predicted to be associated with relatively slower senescence rates. This phenomenon has been demonstrated to occur in laboratory experiments on model species where a reduction in calories (Ozanne and Hales 2004), or protein (Aihie Sayer et al. 2001; Langley‐Evans and Sculley 2006), during development has negative effects on late‐life fitness. However, Lemaître et al. (2015) reported that trade‐offs between early‐life reproduction and late‐life reproduction are frequently observed in the wild across many taxa. If a high‐quality environment during early‐life results in higher rates of early‐life reproduction, then the existence of such trade‐offs leads to a prediction that favorable early environmental conditions should be associated with faster rates of senescent decline in late life. This phenomenon of trade‐offs has recently been demonstrated to occur in lab‐based experiments of arthropods (Adler et al. 2016; Hooper et al. 2017).

Recent reviews have highlighted the uncertainty in our current understanding of the influence of the developmental environment on late‐life fitness. For example, there is clear mechanistic evidence that female gonads are incredibly sensitive to nutritional perturbations during development (Gunn et al. 1995; Rae et al. 2001). However, despite this, there is no clear evidence that nutrition during development influences lifetime reproductive performance, or reproductive senescence onset in female domestic livestock (reviewed in Gardner et al. 2008). A cross‐taxa meta‐analysis found no evidence that experimentally restricted nutrition during development affects longevity overall, although the effect changes direction depending on taxonomic group, and whether nutrition limitation occurs pre‐ or postnatally (English and Uller 2016). A follow‐up meta‐analysis found that, in some circumstances, dietary restriction during development increases interindividual variability in longevity, suggesting that nutritional stress during development might increase phenotypic variation, for example through exposing cryptic epigenetic variation (Senior et al. 2017). These results highlight the complexity of the potential impact of the developmental period on late‐life, and emphasize the need for a review to test whether silver‐spoon effects persist to affect senescence rates in the last stage of life.

Here, we provide, to our knowledge, the first quantitative review of the effect of environmental conditions experienced during the developmental period on senescence rates in wild populations. Using data from longitudinal studies of wild animal populations, we amalgamated effect sizes through meta‐analyses to determine if there is a global mean effect of the quality of the developmental environment on rates of (i) survival senescence, and (ii) reproductive senescence. We then investigated the influence of study characteristics and biological factors in moderating the observed effects.

## Methods

### LITERATURE SEARCH

We performed a broad search of the scientific literature in order to identify published tests of the effect of developmental environment on rates of senescence. For a detailed account of the parameters and methods involved in our literature search, see Appendix S1. In brief, we first used *Web of Science* to identify relevant articles by conducting a key word search with the terms: (senescence OR ageing OR aging) *AND* (“early environment*” OR “early*life” OR “natal environment*” OR “silver*spoon” OR “predictive adaptive response” OR “cohort effect*”). We then conducted forward and backward searches on six classic senescence articles as well as five recently relevant and influential review articles (see Appendix S1 for details). This resulted in a pool of 6766 unique articles. From this pool, we extracted all articles that met the criteria that the study should: (i) use individual‐level data from a population of wild animals to investigate the effect of the environment experienced during development on senescence rates in either survival probability and/or reproductive output (ii) include one or more linear model(s) testing the effect of an interaction between age (measured as a value of discrete time periods, typically years) and a measure of developmental environmental quality, on senescence rates (measured in the same units of time as “age”); (iii) use a metric of environmental quality that was extrinsic to the individual, but that could theoretically have a causal influence on the fitness of the individual. Examples of suitable metrics of environmental quality included weather variables, conspecific population density, food availability, or predation risk. As we were interested in the influence of environmental conditions experienced during development, we only included articles that quantified the metric of environmental conditions during the period of gestation or the period of juvenility (defined as before sexual maturity).

From the published material, we identified 10 articles that measured the effect of developmental environment on senescence rates as per our inclusion criteria. Several additional articles from our initial search pool indicated the availability of the data necessary to complete the required analysis, but did not present results in such a way that they met our criteria. In these cases, we contacted the authors to enquire about the relevant analyses for their study system. From this, we obtained additional results for four species that were previously unpublished.

### DATA SYNTHESIS

In total, we extracted 48 effect sizes from 14 independent studies of six bird and eight mammal species (Table 1). For seven species, 18 effect sizes predicted survival senescence rates, and for 13 species, 30 effect sizes predicted reproductive senescence rates. For the survival senescence models, the response variable was the probability of survival over a given period (typically a year) at a given age. For the reproductive senescence models, the predictor variable was either the probability of reproducing (yes/no), or the magnitude of reproductive output (number of offspring) over a given period (typically a year) for a given age, depending on the biological suitability of either of these measures for the specific study population.

**Table 1:** Summary of the 14 studies included in meta‐analyses

Species	Reference	*N* *	Environmental metrics tested for influence on reproductive senescence	Environmental metrics tested for influence on survival senescence
Barn swallow, *Hirundo rustica*	Balbontín et al. 2015	96 (female and male)	Population density, predation	—
White stork, *Ciconia ciconia*	Baos et al. (2012)	111 (female), 138 (male)	Acute pollution exposure	—
Great tit, *Parus major*	Bouwhuis et al. (2010)	488 (female)	Population density, birthdate, population average reproductive success, number of siblings, food availability, maternal age	—
Mauritius Kestrel, *Falco punctatus*	Cartwright et al. (2014)	52 (female)	Habitat quality	—
Tawny owl, *Strix aluco*	Millon et al. (2011)	40 (female), 88 (male)	Food availability	—
Seychelles Warbler, *Acrocephalus sechellensis*	Hammers et al. (2013)	270 (female and male)	—	Population average reproductive success, food availability, social group size
Svalbard Reindeer, *Rangifer tarandus*	Douhard et al. (2016)	157 (female)	Weather (precipitation)	Weather (precipitation)
Soay sheep, *Ovis aries*	Hayward and Pemberton (2015; unpublished)	447 (female), 130 (male)	Population density	Population density
Red deer, *Cervus elaphus*	Nussey et al. (2007)	214‐253 (female)	Population density	Population density
Mountain goat, *Oreamnos americanus*	Panagakis, Hamel, and Côté (2017)	142 (female)	Population density	Population density
Bighorn sheep, *Ovis canadensis*	Pigeon and Festa‐Bianchet (2017; unpublished)	140 (female)	Population density, weather (mean precipitation), weather (pacific decadal oscillation), weather (mean temperature)	Population density, weather (mean precipitation), weather (pacific decadal oscillation), weather (mean temperature)
Red squirrel, *Tamiasciurus hudsonicus*	Hämäläine, Haines, and Boutin (2017; unpublished)	102 (female), 71 (male)	Food availability	—
Banded mongoose, *Mungos mungo*	Marshall and Cant (2017; unpublished)	13‐53 (female), 11–34 (male)	Weather (variation in rainfall), weather (mean rainfall)	Weather (variation in rainfall), weather (mean rainfall)
Asian elephant, *Elephas maximus*	Mumby et al. (2015)	455 (female)	Weather (mean rainfall)	—

^*^ *N* = the number of individuals used in a study.

Since the biological relevance of different measures of environmental quality will vary across species, it is unsurprising that a broad variety of indices of environmental quality was used. Across studies, conspecific population density was the most widely used metric (six out of the 14 studies, generating 11 effect size), whereas across effect sizes, weather conditions were the most frequently used (21 out of the total 48 effect sizes). Direct measures of food availability accounted for the third most common metric (six effect sizes from four studies). All the other metrics used to measure environmental quality, including acute pollution exposure, birth date, cohort birth‐rate, social group size, anthropogenic habitat exposure, maternal age, sibling number, and predation risk, were each used in only one or two studies, generating one or two effect sizes each.

We followed the predictions of the authors in assigning the directionality of each measure of environment to represent either “better” or “worse” environmental quality. Increasing numerical values of some environmental measures were inferred to represent increasing environmental quality (e.g., food availability), whereas others implied decreasing environmental quality (e.g., population density). To standardize effect sizes, we corrected the direction of each effect size so that the increasing numerical value of the metric of environmental quality reflected increasing environmental quality.

We further categorized effect sizes by several study‐design and biological factors: *type of environmental measure*, *class*, *time of environmental measure*, and *sex*. Due to the large variety of measures of environmental quality, we did not have appropriate statistical power to determine the independent influence of all 11 measures. Instead, we categorized *type of environmental measure* into three groups: measures of population density (*density*), measures related to weather variables (*weather*), and all other measures (*other*; see above). *Class* was categorized as either *mammal* (32 effect sizes from eight species), or *bird* (16 effect sizes from six species). We categorized *time of environmental measure*, as *gestation* if the measure of the environment was taken before birth (eight effect sizes from three species), or *juvenility* if the measure of the environment was taken within or at the first year of life (40 effect sizes from 12 species). *Sex* had three categories (*female*, 35 effect sizes from 13 species; *male*, eight effect sizes from five species, or *both*, five effect sizes from two species). We later reduced analysis of *sex* to only two categories (*female* and *male*; see methods–‐meta‐analysis below).

Following the formulae listed in Koricheva et al. 2013, we transformed each interaction term between developmental environment and age (i.e., the effect size which predicted senescent decline) into a correlation coefficient (*r*) using the relevant inference (test) statistic (i.e., *Χ^2^*, *F*, *T*, or *z*). Then, to avoid a skewed distribution near *r* = ±1, we transformed correlation coefficients with Fisher's z‐transformation (Koricheva et al. 2013). We used these z‐transformed correlation coefficients (*Z_r_*) as standardized effect sizes in all meta‐analyses (see below). We calculated the variance for each effect as 1/(N−3), where *N* is the number of individuals in the analysis (Koricheva et al. 2013).

When inference statistics were not adequately reported in published materials, we contacted authors to request these metrics. In the single case in which an author was unable to reproduce the necessary statistics, we conservatively estimated the nonsignificant effect size as 0 (following Nakagawa and Santos 2012).

### MULTILEVEL META‐ANALYSES

We conducted two separate random effects multilevel meta‐analyses (Konstantopoulos 2011; Koricheva et al. 2013) to determine the mean effect (β_mean_) of developmental environment on both reproductive senescence rates and survival senescence rates. We used a multilevel structure to control for potential nonindependence of effect sizes. Studies contributed multiple effect sizes to either models of reproductive or survival senescence if they contained analyses of both males and females, modeled more than one measure of environmental quality, or modeled environmental quality over each of the time periods (*gestation* and *juvenility*). Within studies, a single model also contributed multiple effect sizes if different measures of environmental quality were analyzed within the same model. To control for potential nonindependence within studies and within models, we ran three‐level meta‐analyses with a random effect of *model*, nested within a random effect of *study* (Konstantopoulos 2011; Nakagawa and Santos 2012; Koricheva et al. 2013). By controlling for study, we did not need to control for nonindependence within species as each species was represented by only one study. To estimate residual variance, we added an observation‐level random effect (Harrison 2014).

After determining the global mean effect on rates of survival senescence and reproductive senescence, we used meta‐regressions to investigate if the mean effect sizes varied dependent on each biological or study‐design factor (see “Data synthesis”). Due to the relatively small sample of effect sizes, each categorizing factor (i.e., *type of environmental measure*, *class*, *time of environmental measure*, and *sex*) was investigated independently as the single categorical moderator term in a meta‐regression. For the meta‐regression with moderator *sex*, we removed effect sizes that considered both sexes due to small sample size (three effect sizes for survival senescence, and two effect sizes for reproductive senescence). For each meta‐regression, we determined if the mean effects of categories were different from each other using a Wald‐type chi‐square test (*Q_m_*). We also determined the mean effect for each category (β) and tested if the effect within each category was different than 0 using a z‐test.

### HETEROGENEITY AND SENSITIVITY ANALYSES

To quantify heterogeneity between effect sizes caused by differences between studies and differences within studies, we calculated *I^2^* statistics. *I^2^* is the ratio of true heterogeneity to the total variance (true heterogeneity plus sampling error) among effect sizes (Koricheva et al. 2013). Due to the codependence in our meta‐analysis models, and resulting use of *study* and *model* as nested random effects, along with an observation‐level random effect, it was necessary to use a modified *I^2^* statistic appropriate for multilevel models (Nakagawa and Santos 2012). For each of the two meta‐analyses, we calculated three *I^2^* values, one attributable to the proportion of true variation between studies, the second attributable to the proportion of true variation within studies (i.e., between models within the same study), and the third attributable to residual variation. The sum of the percentages of total variation due to these three sources equals the traditional *I^2^* of Higgins et al. (2003). By convention, benchmarks for *I^2^* of 25, 50, and 75% are used to indicate low, moderate, and high values of inconsistency between studies (Higgins et al. 2003).

Biases within a dataset used for meta‐analyses can result from a multitude of factors, including unequal research attention across taxonomy, publication biases toward significant results or predicted outcomes, or limitations in the comprehensiveness of literature searches methods (Koricheva et al. 2013). To investigate the potential for biases in our dataset we first created funnel plots and performed Egger's regressions on residuals of the individual effect sizes for each meta‐analysis (Egger et al. 1997; Nakagawa and Santos 2012). Second, we added year of publication as a moderator variable to analyses to determine if there had been a shift in the mean reported effect with time (β_publication year_). Since our dataset included both published and unpublished effect size estimates, we also conducted bias detection tests on the subset of effect sizes from published materials to differentiate apparent publication bias from other causes of bias.

Although it was unavoidable in the context of this study and the available published data, deriving effect sizes from inference statistics rather than bivariate correlations has the potential to introduce inaccuracies in estimates of *Z_r_* (Aloe 2015). The problem may be further amplified by increasing covariance with covariates in the model from which the focal effect test statistic is derived (Aloe 2015; Noble et al. 2017). To investigate the possibility of covariates altering the magnitude or direction of effect sizes, we added the number of covariates as a moderator variable to analyses. For detailed discussion on the potential shortcomings associated with using partial correlation coefficients in lieu of bivariate correlations, readers are encouraged to see Aloe (2015) and Noble et al. (2017).

As an additional potential shortcoming of using partial effect sizes to derive estimates of *Z_r_*, nonindependence between measurements may also influence the estimate of effect (Nakagawa and Cuthill 2007; Noble et al. 2017). Sources of nonindependence can be controlled for by adding random effects into the model from which an effect size is derived (Nakagawa and Santos 2012). Since individuals are measured repeatedly (i.e., annually), having *individual ID* as a random effect in the models from which we derived effect sizes is important to control for this source of nonindependence between measurements. For 44 of the 48 derived effect sizes, the original model included *individual ID* as a random effect. We reran our models excluding the four studies that did not include *individual ID* and this did not change any interpretations of results. Other sources of nonindependence, including similarities in space (e.g., region), time (e.g., cohort, current year), and social structure (e.g., group ID) were also sometimes, but not always, controlled for in the primary source models as random effects (Appendix S2). Since the importance of these various factors as sources of nonindependence will depend on the specifics of each individual study system, we did not make any assumptions about whether or not the inclusion of these random effects was necessary to each individual study. It is important to note however, that if sources of nonindependence were not adequately accounted for in the models from which effect sizes were extracted, then this could influence our results by falsely inflating *N* and reducing the variance as it was calculated.

The full dataset is provided in Appendix S2. All statistical analyses were conducted in R (R Core Development Team 2017, version 3.3.3) with the *metafor* package (Viechtbauer 2010).

## Results

**Figure 1:**
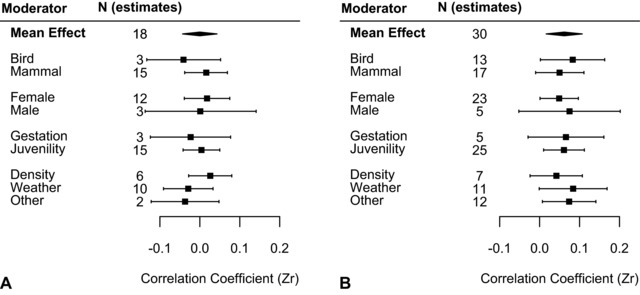
The overall mean effect and the marginal mean effects (with associated 95% CIs) for each of the meta‐regressions categories (*class, sex, time of environmental measure, and type of environmental measure*) for (A) survival senescence rates and (B) reproductive senescence rates.

We found no effect of developmental environment on survival senescence [β _mean_ = –1.2 × 10^−4^, 95% confidence interval (CI) = –0.043 to 0.043, Table 2, Fig. 1A]. However, there was a positive effect on late‐life reproductive success (β _mean_ = 0.062, 95% CI = 0.016 to 0.107, Table 3, Fig. 1B), indicating that, on average, a better quality developmental environment was associated with slower rates of reproductive senescence.

**Table 2:** Results from the random‐effects meta‐analyses on rate of survival senescence

Moderator	*Q_m_* *	*d.f*. (*Q_m_*)	*P* (*Q_m_*)	Category	*m* †	*k* ‡	Mean (*Z_r_*)	Standard error	Lower CI (2.5%)	Upper CI (97.5%)	*z* value	*P (z)*
Mean effect	—	—	—	—	7	18	−0.0001	0.022	−0.043	0.043	−0.005	0.996
Type of environmental measure	2.377	2	0.305	Density	5	6	0.026	0.028	−0.028	0.080	0.939	0.348
				Weather	3	10	−0.029	0.032	−0.091	0.033	−0.919	0.358
				Other	1	2	−0.037	0.043	−0.122	0.048	−0.851	0.395
Class	1.070	1	0.301	Bird	1	3	−0.041	0.048	−0.135	0.052	−0.865	0.387
				Mammal	7	15	0.016	0.027	−0.038	0.069	0.571	0.568
Time of environmental measure	0.250	1	0.617	Gestation	2	3	−0.023	0.051	−0.124	0.077	−0.455	0.649
				Juvenility	6	15	0.004	0.004	−0.042	0.050	0.171	0.864
Sex	0.045	1	0.831	Female	6	12	0.018	0.029	−0.039	0.075	0.620	0.535
				Male	2	3	0.001	0.077	−0.149	0.151	0.016	0.988

^*^ *Q_m_* = Wald‐type chi‐square distribution test statistic for the effect sizes between categories (95% CI).

^†^ *m* = number of species.

^‡^ *k* = number of effect sizes.

No effects are significant.

**Table 3:** Results from the random‐effects meta‐analyses on rate of reproductive senescence

Moderator	*Q_m_* *	*d.f*. (*Q_m_*)	*P* (*Q_m_*)	Category	*m* †	*k* ‡	Mean (*Z_r_*)	Standard error	Lower CI (2.5%)	Upper CI (97.5%)	*z* value	*P (z)*
**Mean effect**	**—**	**—**	**—**	**—**	**13**	**30**	**0.062**	**0.023**	**0.016**	**0.107**	**2.666**	**0.008**
Type of environmental measure	1.288	2	0.525	Density	6	7	0.042	0.033	−0.024	0.107	1.251	0.211
				Weather	4	11	0.084	0.043	−0.001	0.169	1.936	0.053
				**Other**	**6**	**12**	**0.074**	**0.034**	**0.007**	**0.141**	**2.175**	**0.030**
Class	0.397	1	0.529	**Bird**	**5**	**13**	**0.083**	**0.041**	**0.002**	**0.163**	**2.018**	**0.044**
				Mammal	8	17	0.050	0.031	−0.010	0.111	1.623	0.105
Time of environmental measure	0.009	1	0.924	Gestation	3	5	0.066	0.049	−0.029	0.161	1.362	0.173
				**Juvenility**	**11**	**25**	**0.061**	**0.026**	**0.010**	**0.112**	**2.357**	**0.018**
Sex	0.151	1	0.698	**Female**	**12**	**23**	**0.049**	**0.025**	**0.001**	**0.097**	**1.982**	**0.048**
				Male	4	5	0.075	0.065	−0.052	0.202	1.161	0.246

^*^ *Q_m_* = Wald‐type chi‐square distribution test statistic for the effect sizes between categories (95% CI).

^†^ *m* = number of species.

^‡^ *k* = number of effect sizes.

Significant mean effects are bold.

Heterogeneity between effect sizes not accounted for by sampling error, *I^2^*, was low for reproductive senescence (*I^2^_total_* = 37%, *I^2^_between‐studies_* = 25%, *I^2^_within‐studies_* = 12%, residual variance < 0.1%) and negligible for survival senescence (*I^2^_total_* < 0.1%).

The results of the two meta‐analyses were robust to Egger's regression bias detection tests. We detected a significant bias toward positive results from the subset of effect sizes extracted from published material (*t*_21_ = 2.19, *P* = 0.04, Table S1, Fig. S2), but not in models of the complete dataset, in which there was no significant asymmetry in funnel plots for survival senescence (*t*_16_ = 0.58, *P* = 0.57, Table S1, Fig. S2A), or reproductive senescence (*t*_18_ = 1.31, *P* = 0.20, Table S1, Fig. S2B). There was no effect of adding the year of publication of the study (or time of acquirement for unpublished data) as a moderator variable in meta‐regressions in either the model of survival (β_publication year_ = 0.008, 95% CI = −0.032 to 0.047) or reproductive senescence (β _publication year_ = –0.006, 95% CI = –0.045 to 0.032), indicating no discernable shift in results with time.

The number of covariates in the model from which each effect size was derived had no effect on Z_r_ for either estimates predicting reproductive senescence rates (β_number of covariates_ = 6.1 × 10^−5^, 95% CI = –0.009 to 0.009), or survival senescence rates (β _number of covariates_ = –0.005, 95% CI = –0.016 to 0.007).In the meta‐regressions of both the effect on survival and reproductive senescence rates, there were no significant differences in mean effect between categories (P(*Q_m_*) values, Tables 1 and 2).

## Discussion

Our meta‐analysis of eight mammal and six bird species indicated that variation in early environmental conditions affected rates of reproductive senescence, but found no evidence of effects of early environment on survival senescence.

### REPRODUCTIVE SENESCENCE

The significant mean effect of developmental environment on reproductive senescence indicates that the environmental conditions during development have far‐reaching effects all the way into the last stage of life. The positive direction of this effect implies that good developmental conditions provide a silver‐spoon, whereby the rates of decline of reproductive output in late life are slower when the quality of the environment experienced during development is better.

Although the effect of developmental environment on reproductive senescence was significant, the effect size was relatively small, as per the conventional benchmarks proposed by Cohen (1988). This is not entirely surprising given the multitude of complex and potentially interacting environmental and genetic factors that are expected to influence senescence.

In an evolutionary context, we assume that senescence is partially genetically determined (Kirkwood and Austad 2000; Patridge and Gems 2007; Nussey et al. 2013; Kowald and Kirkwood 2015; Reichard 2016), and several studies have confirmed the heritability of individual senescence patterns (Arking 1987; Charmantier et al. 2006; Wilson et al. 2007; Wang et al. 2014). The effect of developmental environment on reproductive senescence may therefore represent a “genotype by past‐environment” interaction. This plasticity in senescence rate will weaken the ability of the trait to respond to selection, and thus might even act as a mechanism by which senescence persists in the wild.

Although in some study systems an individual's developmental environment may be correlated with their late‐life environment, we believe it is unlikely that the effect of development environment on reproductive senescence is driven by such a correlation. For four of the species in our dataset (great tit, Svalbard reindeer, red deer, and Soay sheep) current environmental conditions were controlled for in the regression models from which effect size was extracted. For Mauritius kestrels, the environmental measure used had no association with fitness when measured in adults (Cartwright et al. 2014), indicating that the measure of environmental quality used only had biological relevance during the highly sensitive developmental life stage. For other species, the environmental measures selected cycled from low to high multiple times within the lifespans of individuals that reached the age where senescence became apparent (prey density in tawny owls; Lambin et al. 2000, and monsoon season in Asian elephants; Mumby et al. 2015), were highly stochastic within the study system they were used (spruce masting in red squirrels; Boutin et al. 2006), or necessarily changed between development and adulthood due to breeding dispersal (territory quality in Seychelles warblers; Hammers et al. 2013). In these cases, a correlation between developmental and late‐life environment is unlikely. It is also worth noting that since trade‐offs within individuals between early‐adult and late‐life reproduction have been demonstrated to occur commonly in wild populations (Lemaître et al. 2015), our tests may actually be conservative. This is because if good environmental conditions during the first part of life (i.e., during development and early‐reproductive life) result in higher reproductive investment in early adult life, investment in late‐life reproduction should be reduced, potentially increasing the rates of reproductive senescence. In addition, if a correlation between developmental and late‐life environment was what was driving the effect of developmental environment on senescence, we would expect to see a significant effect in survival senescence rates (Reichert et al. 2010; Bleu et al. 2015; Le Coeur et al. 2016; Péron et al. 2016), in addition to the observed effect on reproductive senescence rates.

The low level of heterogeneity amongst effect sizes (*I^2^_Reproduction (total)_* = 37%) indicates that there may be some differences in the true effect of developmental environment on reproductive senescence rates based on biological or study design factors. Although none of the meta‐regressions found significant differences between the categories within moderator terms (*type of environmental measure*, *class*, *time of environmental measure*, and *sex*), there were some suggestive trends. The reproductive senescence of birds, but not mammals, was significantly affected by developmental environment. Additionally, environmental measures of weather had roughly twice the magnitude of effect in predicting reproductive senescence rates as compared to environmental measures of population density. Although there were apparent differences between categories in *sex* (significant effect in females, but not males) and *time of environmental measure* (significant effect of measures taken during juvenility, but not gestation), in these two cases the differences were likely driven by the relatively small sample sizes available for the nonsignificant categories.

### SURVIVAL SENESCENCE

The lack of a perceptible effect of developmental environment on survival senescence was uniform across studies and meta‐regression categories. This uniformity in nonsignificance was likely a driver of the negligible heterogeneity seen between effect sizes (*I^2^_Survival (total)_* < 0.1%). However, the *I^2^* statistic is known to have low power when sample sizes are small (Huedo‐Medina et al. 2006; Nakagawa and Santos 2012; Koricheva et al. 2013), and so the relatively small sample size may be contributing to the low *I^2^* value.

It is possible that a silver‐spoon effect does actually slow survival senescence, but that the effect is masked by selective disappearance, directly caused by differences in environmental quality experienced during development (often referred to as the “selection hypothesis”; Nol and Smith 1987; Dugdale et al. 2011). Ability to survive into old age may be causally linked with ability to withstand harsh extrinsic conditions. Thus, stronger selection pressure for survival‐related traits could result in a greater average ability to resist senescence among those that developed under poor conditions, concealing any silver‐spoon effect on survival senescence rates.

Alternatively, the lack of effect on survival senescence rates may result from adaptive physiological mechanisms present during development that primarily conserve survival‐enhancing traits at the cost of reproduction‐enhancing traits. When available energy is limited by poor environmental conditions, trade‐offs that favor early‐life survival over early‐life reproductive development may be expected to occur (Kirkwood and Rose 1991). Thus, there is a carry‐over cost of reduced reproductive ability when developmental environment is poor, but a relatively lower carry‐over cost to survival ability. As a result, survival senescence may be less plastic than reproductive senescence, being less influenced by variability in the extrinsic environment during development.

### FUTURE DIRECTIONS

Several questions remain regarding the influence of developmental environment on senescence rates. Most notably, we found no relevant studies of amphibians, reptiles, fishes, or any invertebrates, which is presumably reflective of the relative lack of long‐term individual‐based studies of these species in the wild (Clutton‐Brock and Sheldon 2010). Given the inherent physiological differences (Wang et al. 2006), commonality of indeterminate growth (Kozlowski 1996), and slower or absent senescence patterns (Flouris and Piantoni 2015) in ectotherms, it is plausible the environment experienced during development may have vastly different mechanistic influences on senescence (Hooper et al. 2017, 2018). However, due to the lack of long‐term studies, effects remain principally unknown. Additionally, our results are suggestive of differences between birds and mammals, as well as between different measures of developmental environment (categorized as *density*, *weather*, or *other*) on the magnitude of effect observed in reproductive senescence rates. A greater number of studies would allow for a more robust test of the biological significance of these trends.

Through our literature search, we identified several studies with data that would have been adequate to investigate this question, but the specific analyses had not been done. In the hope of encouraging a more extensive synthesis in the future, we have formulated some suggestions for specific analytical methods to investigate the influence of developmental environment on senescence in wild populations. We recommend that: *(i)* To differentiate the influence of environmental quality during development and during other periods of life‐history, the measure of environmental quality should be contained to only some predefined period, ending before sexual maturity. *(ii)* The effect should be analyzed using a model predicting either survival probability or reproductive output within a discreet time sequence (i.e., annually). As a predictor term, age should interact with the specific measure of developmental quality to specifically determine an effect on senescence rates. *(iii)* These models must only include individuals above the age of the onset of senescence (i.e., once reproductive or survival probability begins to decline), otherwise, any perceivable effect may be reliant on altering survival or reproductive rates in another period of life. *(iv)* Models with repeat measures of individuals should include individual ID as a random effect. An effort to control for other expected codependences through time and space should be made where ever possible (e.g., a random effect of year if fitness varies considerably with year). *(v)* Metrics of environmental quality should be chosen with care, and ideally, a metric should be demonstrated to scale linearly with fitness before being used.

We hope that publication of additional studies, and in particular, across other taxa, will further clarify our understanding of the long‐term effects of developmental environment on late‐life performance.

Associate Editor: A. Charmantier

## Supporting information


**Table S1**. Eggers regression models for the subset of effect estimates from the survival senescence model, the reproductive senescence model, and all published effect sizes (reproductive and survival) combined.
