# Supplementary material for: Ageing with a silver‐spoon: A meta‐analysis of the effect of developmental environment on senescence
Source: Evol Lett. 2018 Aug 16;2(5):460–71. doi: 10.1002/evl3.79 (PMC6145406; doi:10.1002/evl3.79)
Supplement: Supplementary file 1 — Table S1. Eggers regression models for the subset of effect estimates from the survival senescence model, the reproductive senescence model, and all published effect sizes (reproductive and survival) combined. [file EVL3-2-460-s005.docx]

**Table S1:** Eggers regression models for the subset of effect estimates from the survival senescence model, the reproductive senescence model, and all published effect sizes (reproductive and survival) combined.

| Effect estimates | *K* | *t* | *d.f.* | *p* |
| --- | --- | --- | --- | --- |
| Survival | 18 | 0.579 | 16 | 0.571 |
| Reproductive | 30 | 1.311 | 28 | 0.200 |
| **Published** | **23** | **2.187** | **21** | **0.040** |
