# Supplementary figures and images for: Ageing with a silver‐spoon: A meta‐analysis of the effect of developmental environment on senescence
Source: Evol Lett. 2018 Aug 16;2(5):460–71. doi: 10.1002/evl3.79 (PMC6145406; doi:10.1002/evl3.79)

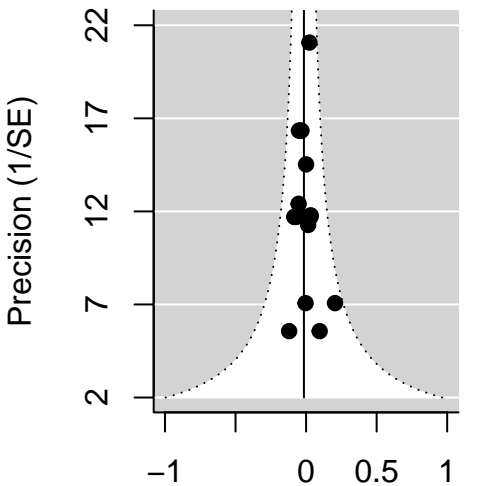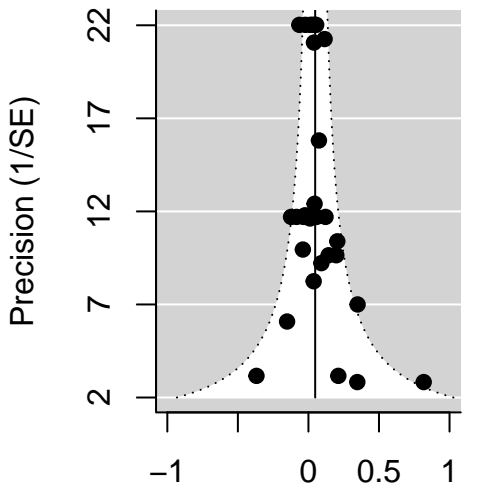

Supplement: Supplementary file 2 [file EVL3-2-460-s001.pdf]

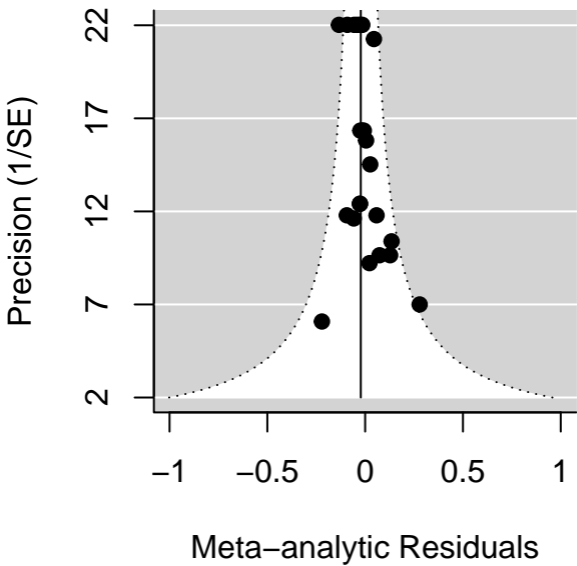

Supplement: Supplementary file 3 [file EVL3-2-460-s002.pdf]
