# Supplementary material for: Ageing with a silver‐spoon: A meta‐analysis of the effect of developmental environment on senescence
Source: Evol Lett. 2018 Aug 16;2(5):460–71. doi: 10.1002/evl3.79 (PMC6145406; doi:10.1002/evl3.79)
Supplement: Supplementary file 4 [file EVL3-2-460-s003.docx]

**Appendix S1**

**Literature Search**

All parts of the literature search were conducted using *Web of Science* between May and September 2017. We included results from all years to present, and included the databases: Science Citation Index Expanded (SCI-EXPANDED; 1945-present), Social Sciences Citation Index (SSCI; 1956-present), Arts & Humanities Citation Index (A&HCI; 1975-present), Conference Proceedings Citation Index- Science (CPCI-S; 1990-present), Conference Proceedings Citation Index- Social Science & Humanities (CPCI-SSH; 1990- present), Current Chemical Reactions (CCR-EXPANDED; 1985-present, *Includes Institut National de la Propriete Industrielle structure data back to 1840*), and Index Chemicus (IC; 1993-present).

With these databases, we first conducted a key-word search with the terms: Topic = (senescence OR ageing OR aging) *AND* Topic ("early environment*" OR "early*life" OR "natal environment*" OR "silver spoon" OR "predictive adaptive response" OR "cohort effect*"). Following the search protocol of the senescence review paper by Lemaître et al. (2015), we then conducted forward searches on six ‘classic’ papers on the evolution of ageing:

Hamilton, W.D. (1966). The moulding of senescence by natural selection*. J. Theor. Biol.,* 12, 12–45.

Kirkwood, T.B. (1977). Evolution of ageing. *Nature,* 270, 301–304.

Kirkwood, T.B & Holliday, R. (1979). The evolution of ageing and longevity. *Proc. R. Soc. Lond. B.,* 205, 531–546.

Kirkwood, T.B. & Rose, M.R. (1991). Evolution of senescence: late survival sacrificed for reproduction. *Phil. Trans. R. Soc. Lond. B.,* 332, 15–24.

Kirkwood, T.B. & Austad, S.N. (2000). Why do we age? *Nature,* 408, 233–238.

Williams, G.C. (1957). Pleiotropy, natural selection, and the evolution of senescence. *Evolution,* 11, 398–411.

We also conducted backwards and forwards searches on the following five recent or influential review papers related to senescence or the influence of early life on subsequent life-history:

Lindström, J. (1999). Early development and fitness in birds and mammals. *Trends Ecol. Evol.,* 14, 343–348.

Lemaître, J-F., Berger, V., Bonenfant, C., Douhard, M., Gamelon, M., Plard, F., Gaillard, J-M. (2015). Early-late life trade-offs and the evolution of ageing in the wild. *Proc. R. Soc. B.,* 282: 20150209.

Lemaître J-F. & Gaillard, J-M. (2017). Reproductive senescence: new perspectives in the wild. *Biol. Rev.,* 92, 2182-2199.

Monaghan, P. (2008). Early growth conditions, phenotypic development and environmental change. *Phil. Trans. R. Soc. B.,* 363, 1635-1645.

Nussey, D.H., Froy, H., Lemaître, J-F., Gaillard, J-M., Austad, S.N. (2013). Senescence in natural populations of animals: Widespread evidence and its implications for bio-gerontology. *Ageing Res. Rev.,* 12, 214-225.

In total, the above searches yielded 6766 unique results (see endnote library). Titles, abstracts, and, where necessary, paper bodies were reviewed by E.C. to identify relevant papers under the inclusion criteria (see below). By this method, 10 papers containing the required analysis were found. Two of these papers did not report the necessary statistics on some non-significant results, which were needed to calculate correlation coefficients. In both these cases, we contacted authors directly and they were able to provide us the neccessary information. Through the aide of Raquel Baos, the unpublished statistics required for white storks (*Ciconia ciconia*) was acquired. Dan Nussey provided statistics necessary to calculate the effect of density on survival senescence rates in red deer (*Cervus elaphus*).

For additional papers we found that demonstrated the availability of data necessary to complete the required analysis, but did not present results in such a way that they met our parameters, we contacted the primary authors to enquire about the relevant analyses for their study system. From this, we obtained additional analyses that were previously unpublished. Analyses were provided by Alexandre Millon (tawny owls; *Strix aluco*), Harry Marshall (banded mongooses; *Mungos mungo*), Gabriel Pigeon (bighorn sheep; *Ovis canadensis*), Anni Hämäläinen and Jessica Haines (red squirrels; *Tamiasciurus hudsonicus*). Additionally, Adam Hayward provided the raw data used in analysis from a 2015 paper on Soay sheep (*Ovis aries*; Hayward *et al.*, 2015, *Exp. Gerontol.,* 71, 56-68). From this raw data, E.C. calculated the 2 survival and 1 reproductive senescence rate effect sizes included for this species.
